# Estimation of Hazard Functions in the Log-Linear Age-Period-Cohort Model: Application to Lung Cancer Risk Associated with Geographical Area

**DOI:** 10.4137/cin.s4522

**Published:** 2010-04-14

**Authors:** Tengiz Mdzinarishvili, Michael X. Gleason, Simon Sherman

**Affiliations:** Eppley Cancer Institute, University of Nebraska Medical Center, 986805 Nebraska Medical Center, Omaha, NE. Email: ssherm@unmc.edu

**Keywords:** cancer incidence, temporal trend, cohort effect, hazard function, lung cancer

## Abstract

An efficient computing procedure for estimating the age-specific hazard functions by the log-linear age-period-cohort (LLAPC) model is proposed. This procedure accounts for the influence of time period and birth cohort effects on the distribution of age-specific cancer incidence rates and estimates the hazard function for populations with different exposures to a given categorical risk factor. For these populations, the ratio of the corresponding age-specific hazard functions is proposed for use as a measure of relative hazard. This procedure was used for estimating the risks of lung cancer (LC) for populations living in different geographical areas. For this purpose, the LC incidence rates in white men and women, in three geographical areas (namely: San Francisco-Oakland, Connecticut and Detroit), collected from the SEER 9 database during 1975–2004, were utilized. It was found that in white men the averaged relative hazard (an average of the relative hazards over all ages) of LC in Connecticut vs. San Francisco-Oakland is 1.31 ± 0.02, while in Detroit vs. San Francisco-Oakland this averaged relative hazard is 1.53 ± 0.02. In white women, analogous hazards in Connecticut vs. San Francisco-Oakland and Detroit vs. San Francisco-Oakland are 1.22 ± 0.02 and 1.32 ± 0.02, correspondingly. The proposed computing procedure can be used for assessing hazard functions for other categorical risk factors, such as gender, race, lifestyle, diet, obesity, etc.

## Introduction

In cancer epidemiology, a risk of getting a cancer in a given age (*t*) is evaluated by the age-specific incidence rate, *I*(*t*), as the number of cases of a particular type of cancer per 100,000 population. Along with age, race and gender, as well as with time period and birth-cohort effects,^1^–^4^ incidence rates also depend on other risk factors, such as geographical area, dietary factors, life style habits, etc., which can be viewed as categorical variables.

During the last 50 years, finding a direct relationship between the observed incidence rates and risk factors determining these rates has been one of the main challenges of cancer epidemiology. Some progress in solving this problem is achieved by the use of the log-linear model.^5^,^6^ The log-linear age-period-cohort (LLAPC) model is used to account for age, time period and birth-cohort effects.^7^–^10^ According to this model, an age-specific incidence rate of a cancer can be presented as a product of the time period and birth cohort coefficients, as well as an unknown age-specific hazard function, i.e. risk function of getting the cancer at a given age. Recently,^11^ we expanded the use of the LLAPC model on cases when the mathematical form of the hazard function is unknown and proposed a novel computational procedure allowing one to separate the problem of estimating the time period and birth cohort coefficients from the problem of estimating the unknown hazard function.

In the present work, we expand the use of LLAPC model for characterizing unknown hazard functions for populations with different exposures to categorical risk factors (different categories of a categorical variable). In our model, the dissimilarity in exposure is presented by different descriptive categories of the corresponding categorical variable.

The proposed procedure was used for estimating the age-specific hazard functions of lung cancer (LC) for the gender- and race-specific populations living in different geographical areas. For this purpose, we utilized data on LC incidence rates observed in white men and women, in three geographical areas (namely: San Francisco-Oakland, Connecticut and Detroit), collected during 1975–2004. The estimates were obtained from the observed cancer incidence rates, and preliminarily corrected for time period and birth cohort effects. These corrections were made by the approach that we described in.^11^

We have found that the LC hazard functions associated with living in these geographical areas have different amplitudes, but the overall shape of these functions is very similar. We have shown that geographical area risk factors influence the LC age-specific hazard functions in approximately the same manner in all ages.

Thus, in this work we provide a proof-of-concept that the proposed computing procedure can be successfully applied for estimating the influences of categorical risk factors on the hazard functions for a particular type of cancer.

## Materials and Methods

### Log-linear age-period-cohort model

According to the LLAPC model of cancer presentation in aging, the observed incidence rates can be expressed by the product of unknown coefficients of the time period and the birth cohort effects and the unknown hazard function. This function presents a risk to get cancer in aging independently from the time period and birth cohort effects. Until recently, the use of this model in cancer epidemiology was limited to the cases when the mathematical form of the hazard function is known *a priori* (for instance, the form of hazard function can be taken from a biological model of cancer development),^8^ but parameters of this function can be unknown. In this case, the time period coefficients, *v_j_*, the birth cohort coefficients, *u_l_*, as well as parameters of the given hazard function, *h*(*t_i_*), can be derived by solving the following system of conditional equations:

(1) $$I_{i,j}(t_{i})=v_{j}u_{l}h(t_{i});\;i=1,\ldots ,n;j=1,\ldots ,m;l=1,\ldots ,k$$

In (1), *I_i,j_*(*t_i_*) is the observed incidence rate in the *i*-th age interval (*t_i_* denotes the midpoint of this interval) and in the *j*-th time period interval, while index *l* indicates the birth cohort age interval (note, *l* is defined by indices *i* and *j*).^11^ The problem is to derive the time period and birth cohort coefficients, as well as parameters of the hazard function using the incidence rates, observed during the given set of time periods. The main obstacle in solving this problem is that multiple estimators of the time period and birth cohort coefficients can provide equally good solutions.^1^–^4^ It means that for determining these coefficients, the identifiability problem has to be overcome.

In practice, the identifiability problem can be solved by the use of some assumptions. For instance in,^8^ this problem was solved assuming that within each age interval, the observed cancer cases have a Poisson distribution and the mathematical form of the hazard function is given *a priori*. Adjustments of unknown parameters were performed by the LLAPC model using the maximum likelihood method for assessing the birth cohort and time period effect coefficients as well as parameters of the hazard function. An initial assumption that the cohort effect is absent was used at the beginning of the iteration process to determine the birth cohort and time period effect coefficients. These coefficients were estimated by anchoring one time period coefficient (*v* = 1) and one birth cohort effect coefficient (*u* = 1). Thus, the results obtained by this procedure depend on the hazard function used, and on the time period and cohort, to which the coefficients are anchored.

Recently in,^11^ we expanded the use of the LLAPC model of cancer presentation in aging on cases when the mathematical form of the hazard function is unknown. In contrast to the previously used methods, a simple, computationally effective method^11^ provides an estimation of the time period and birth cohort coefficients without any *a priori* knowledge of the hazard function. The only assumption used in that method is that the cohort effect coefficients of the neighbor cohorts are nearly the same. Thus, the results of assessing the birth cohort and time period effect coefficients obtained by the method^11^ depend only on the time period and cohort, to which the coefficients are anchored, but not on the unknown hazard function. It allows one to separate the problem of estimating the time period and birth cohort coefficients from the problem of estimating the unknown hazard function. Moreover, as we have shown below, the use of the procedure^11^ allows one to estimate the age-specific hazard function defined by the certain categorical risk factors.

### Estimation of hazard functions in the LLAPC model

Let us denote by *I_i,j,c_*(*t_i_*) the observed incidence rates of cancer within a population exposed to the given categorical risk factor, presented by a set of descriptive categories (indexes), *c*, of a given categorical variable. In such cases, the LLAPC model can be presented by conditional equations:

(2) $$\begin{array}{l} I_{i,j,c}(t_{i})=v_{j,c}u_{l,c}h_{c}(t_{i})\;i=1,\ldots ,n,\\ \;j=1,\ldots ,m,l=1,\ldots ,k \end{array}$$

Here, *v_j,c_* and *u_l,c_* are the time period and birth cohort effect coefficients for the population exposed to the given category, *c*, of the considered risk factor. In practice, the categories might be encoded as 0, 1, 2, etc.

As can be seen from (2), the hazard function along with the age also depends on the category, *c*. By using our procedure,^11^ one can obtain the estimates of the time period and birth cohort coefficients, $v_{j,c}^{*}$ and $u_{l,c}^{*}$, and their standard errors $\text{SE}(v_{j,c}^{*})$ and $\text{SE}(u_{l,c}^{*})$ (here and below the asterisk denotes estimates, as well as estimators). Again, a distinguishable feature of the procedure^11^ is that the aforementioned estimates are obtained without using any information on the hazard function, *h_c_*(*t_i_*).

Using the obtained estimates of the time period and birth cohort coefficients, $v_{j,c}^{*}$ and $u_{l,c}^{*}$, the observed incidence rates can be corrected for these effects in the following way:

(3) $$\begin{array}{l} I_{i,j,c}^{*}(t_{i})=\frac{I_{i.j,c}(t_{i})}{v_{j,c}^{*}u_{l,c}^{*}};\;\;\;i=1,\ldots ,n;\\ \;\;j=1,\ldots ,m;\;\;\;l=1,\ldots ,k \end{array}$$

In calculations we use only the incidence rates when the number of cases is larger than 15. Therefore, to characterize the error distributions of the incidence rates, the normal distribution (instead of the Poisson distribution usually used) can be utilized.^12^ It can be shown that when coefficients of variation of the *I_i,j,c_*(*t_i_*), $v_{j,c}^{*}$ and $u_{l,c}^{*}$ are small, the incidence rates, $I_{i,j,c}^{*}(t_{i})$, corrected by formula (3), will be normally distributed. This proposition can be proven in the way analogous to one that is presented in^11^ for analyzing the error distribution of the ratio of two observed incidence rates.

According to the standard rules of error propagation, 13 squares of the standard error of $I_{i,j,c}^{*}(t_{i})$, presented by (3), can be calculated by the following formula:

(4) $$\begin{array}{l} {\text{SE}}^{2}[I_{i,j,c}^{*}(t_{i})]={\left(\frac{1}{v_{j,c}^{*}u_{l,c}^{*}}\right)}^{2}{\text{SE}}^{2}[I_{i,j,c}(t_{i})]\\ \;\;\;\;\;\;\;\;\;\;\;\;\;\;\;\;\;\;\;\;\;\;\;\;\;\;\;\;\;\;\;\;\;\;\;\;+{\left[-\frac{I_{i,j,c}(t_{i})}{v_{j,c}^{*2}u_{l,c}^{*}}\right]}^{2}{\text{SE}}^{2}(v_{j,c}^{*})\\ \;\;\;\;\;\;\;\;\;\;\;\;\;\;\;\;\;\;\;\;\;\;\;\;\;\;\;\;\;\;\;\;\;\;\;\;+{\left[-\frac{I_{i,j,c}(t_{i})}{v_{j,c}^{*}u_{l,c}^{*2}}\right]}^{2}{\text{SE}}^{2}(u_{l,c}^{*}) \end{array}$$

where the coefficients before squares of the standard errors are squares of partial derivatives of $I_{c}^{*}$ with respect to *I_c_*, $v_{c}^{*}$ and $u_{c}^{*}$, correspondingly.

From (2) and (3) one can obtain the following system of conditional equations:

(5) $$I_{i,j,c}^{*}(t_{i})=h_{c}(t_{i});\;\;\;i=1,\ldots ,n;\;\;\;j=1,\ldots ,m$$

From (5) it can be seen that for assessing values of the hazard function, *h_c_*(*t_i_*), in each *i*-th age interval there are *m* conditional equations. Therefore, for estimating *n* values (corresponding to the *n* age intervals) of the hazard function there are *n* × *m* conditional equations (5). To solve the system (5), a least squares method can be used.^14^ In such a case, the most efficient estimates for *h_c_*(*t_i_*) can be obtained as the weighted means (averaged through index *j*) of the observed values $I_{i,j,c}^{*}(t_{i})$:

(6) $$h_{c}^{*}(t_{i})=\frac{\sum_{j=1}^{m}w_{i,j}I_{i,j,c}^{*}(t_{i})}{\sum_{j=1}^{m}w_{i,j}}$$

In (6), the weights, *w_i,j_*, are given as reciprocals of the square of the standard error of estimates of the $I_{i,j}^{*}(t_{i})$ given by formula (4). Standard errors of the corresponding estimate, ${\text{SE}}^{2}[h_{c}^{*}(t_{i})]$, can be easily obtained from (6):

(7) $${\text{SE}}^{2}[h_{c}^{*}(t_{i})]=\frac{1}{\sum_{j=1}^{m}w_{i,j}}=\frac{1}{\sum_{j=1}^{m}1/{\text{SE}}^{2}\left[I_{i,j,c}^{*}(t_{i})\right]}$$

(Note, when variables on the left side of the conditional equations (5) are normally distributed with known standard errors, the least square estimators, $h_{c}^{*}(t_{i})$, will be also normally distributed.)

From (3)–(4) and (5)–(6) it follows that estimates, $h_{c}^{*}(t_{i})$, and their *SE* can be calculated by the observed incidence rates, *I_i,j,c_*(*t_i_*), and the estimates of the coefficients, $v_{c}^{*}$ and $u_{c}^{*}$. As noted in,^11^ estimates of the coefficients $v_{j,c}^{*}$ and $u_{j,c}^{*}$ depend on the time period and cohort to which the coefficients are anchored (i.e. on the time period and birth cohort to which adjustments are made). Therefore, for populations differently exposed to the considered risk factor (see below), their hazard functions can be compared only in the cases when the same anchors are used.

### Estimation of the ratio of hazard functions

For populations with different exposures to the considered risk factor, the ratios of the corresponding age-specific hazard functions can be used as a measure of relative hazard. In fact, let us denote by $h_{0}^{*}(t_{i})$ and $h_{1}^{*}(t_{i})$ (*i*= 1,...,*n*) the estimates of the hazard function corresponding to two categories, coded as 0 and 1. Then, at a given age interval, *t_i_*, the ratio, $r_{1|0}^{*}(t_{i})=h_{1}^{*}(t_{i})/h_{0}^{*}(t_{i})$, will present an estimate of the relative hazard for a population coded as *c* = 1 compared to the reference (*c* = 0). Standard errors of the relative hazard, $\text{SE}[r_{1|0}^{*}(t_{i})]$, can be calculated using the $\text{SE}[h_{1}^{*}(t_{i})]$and $\text{SE}[h_{0}^{*}(t_{i})]$ by standard rules of error propagation. The estimate of the averaged relative hazard, $R_{1|0}^{*}$, is calculated by the following formula of weighted mean:

(8) $$R_{1|0}^{*}=\frac{\sum_{i=1}^{n}w_{i}r_{1|0}^{*}(t_{i})}{\sum_{i=1}^{n}w_{i}}$$

In (8), the weights, *w_i_*, are given as reciprocals of the square of the *SE* of estimates of the $r_{1|0}^{*}(t_{i})$. The *SE* of the corresponding estimate, $\text{SE}\left[R_{1|0}^{*}\right]$, can be calculated from the following variance of the weighted mean:

(9) $${\text{SE}}^{2}[R_{1|0}^{*}]=\frac{1}{\sum_{i=1}^{n}w_{i}}=\frac{1}{\sum_{i=1}^{n}1/{\text{SE}}^{2}[r_{1|0}^{*}(t_{i})]}$$

Analogously, taking $h_{0}^{*}(t_{i})$ as a standard, for multiple categories of a given risk factor (coded as *c* = 0, 1, 2, 3, …), the ratios;

(10) $$\begin{array}{l} r_{1|0}^{*}(t_{i})=h_{1}^{*}(t_{i})/h_{0}^{*}(t_{i}),\\ r_{2|0}^{*}(t_{i})=h_{2}^{*}(t_{i})/h_{0}^{*}(t_{i}),\\ r_{3|0}^{*}(t_{i})=h_{3}^{*}(t_{i})/h_{0}^{*}(t_{i}),\\ \ldots \end{array}$$

will give corresponding estimates of the relative hazard at a given age interval, *t_i_*, for populations exposed to the categories, *c* = 0, 1, 2, 3, …, compared to the hazard for a population exposed to the category, *c* = 0. The corresponding averaged relative hazards of exposure to the categories *c* = 1, 2, 3, …, (compared to the hazard of category *c* = 0), i.e. $R_{1|0}^{*}$, $R_{2|0}^{*}$, $R_{3|0}^{*}$, ..., can be calculated by formulas similar to formula (8). Analogously, *SE* of the corresponding estimates (i.e. $\text{SE}\left[R_{1|0}^{*}\right]$, $\text{SE}[R_{2|0}^{*}]$, $\text{SE}[R_{3|0}^{*}]$, ...) can be calculated by formulas similar to formula (9).

## Application

### Estimation of relative risks of lung cancer associated with geographical area

#### Data preparation and processing

As a test-bed for the proposed procedure of evaluation of hazard functions, we analyzed the LC risks associated with a geographical area. In this work, we used the protocol for data preparation, analogous to the one described in.^11^ The first primary, microscopically confirmed LC cases for white men and women collected during 1975–2004 were extracted from the SEER 9 registries. Data for three geographical areas were utilized in our study: (i) San Francisco-Oakland, (ii) Connecticut, and (iii) Detroit, coded as *c* = 0, *c* = 1, and *c* = 2, correspondingly. LC incidence rates, expressed per 100,000 persons, were age-adjusted by the direct method to the 2000 United States standard population.^15^ The *SE* of the age-adjusted incidence rates were calculated as described in.^16^

The obtained incidence rates were grouped in six five-year cross-sectional time periods. These periods were indexed by *j*: 1975–79 (*j* = 1); 1980–84 (*j* = 2); 1985–89 (*j* = 3); 1990–94 (*j* = 4); 1995–99 (*j* = 5); and 2000–04 (*j* = 6). Each of these subsets was grouped into 18 five-year age groups: 17 groups, ranging from 0 to 84 years, and the 18th group that included all cases for ages 85+. These groups were indexed by *i* in the following way: 0–4 (*i* = 1); 5–9 (*i* = 2), 10–14 (*i* = 3), …, 80–84 (*i* = 17), 85+ (*i* = 18). We only used the data for the groups over age 35 (*i* = 8, 9, …, 18), because the incidence rates for these groups had corresponding case counts that were statistically significant. We considered 16 birth cohorts (*l* = 1, 2, …, 16), corresponding to birth year ranges of 1890–94, …, 1965–69.

Thus, the age-adjusted incidence rates of LC in white men (as well as in white women) in three considered geographical areas were presented as the following sets of values: *I_i,j,_*_0_(*t_i_*), *I_i,j_*_,1_(*t_i_*), and *I_i,j_*_,2_(*t_i_*), (*i* = 8, …, 18, *j* = 1, …, 6). Analogously, the *SE* of these incidence rates were presented as:*SE*[*I_i,j_*_,0_(*t_i_*), *SE*[*I_i,j_*_,1_(*t_i_*)], and *SE*[*I_i,j_*_,2_(*t_i_*)] (*i* = 8, …, 18, *j* = 1, …, 6).

## Results and Discussion

Our procedure described in^11^ was used to estimate the time period and birth cohort coefficients (and their *SE*) for the LC age-adjusted incidence rates in white men and women in each of three considered geographical areas. Estimates of the time period and birth cohort coefficients, $v_{j,c}^{*}$ and $u_{l,c}^{*}$ (*c* = 0,1,2), were obtained using $v_{6,c}^{*}=1$ (time period 2000–2004) and $u_{8,c}^{*}=1$ (cohort 1925–1929), as anchors. The estimates, $I_{i,j,c}^{*}(t_{i})$, and their standard errors were obtained by formulas (3) and (4), correspondingly. Finally, estimates of the hazard function, $h_{c}^{*}(t_{i})$, and their *SE* were obtained by formulas (6) and (7).

**Figure 1.**
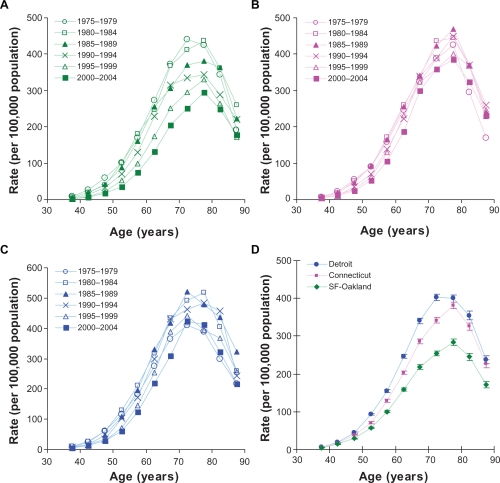
Lung cancer incidence rates in white men during six (five-year) time periods of 1975–2004 in (**A**) San Francisco-Oakland, (**B**) Connecticut, and (**C**) Detroit. (**D**) Estimates of the age-specific hazard functions in these areas (error bars indicate standard error).

**Figure 2.**
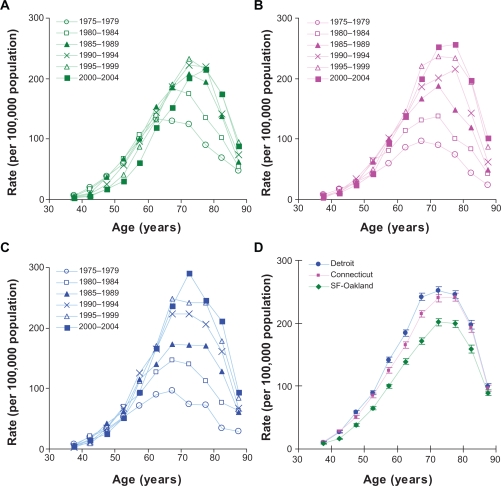
Lung cancer incidence rates in white women during six (five-year) time periods of 1975–2004 in (**A**) San Francisco-Oakland, (**B**) Connecticut, and (**C**) Detroit. (**D**) Estimates of the age-specific hazard functions in these areas (error bars indicate standard error).

Figure 1 presents the incidence rates observed in men during the six (five-year long) time periods of 1975–2004 in San Francisco-Oakland (panel A), Connecticut (panel B), and Detroit (panel C). Panels A–C of Figure 2 present the analogous rates observed in women. As can be seen from the panels A, B and C, the observed incidence rates differ remarkably during the observed six time periods. This significantly complicates studies of relationship between the observed incidence rates and age.

Tables 1 and 2 present the estimates of the age-specific hazard functions (as well as their *SE*) of LC for the considered geographical areas in men and women, correspondingly. Visual presentation of these estimates is given on panels D of Figures 1 and 2. As can be seen from these panels, the distribution of the estimated values of the corresponding hazard functions exhibits definite patterns having common features, such as an exponential rise in values (from the age about 40 until the age about 70), turnover (taking place at the age interval of 70–80) and a fast fall (at the older ages). Interestingly, the absolute values of the hazard functions of LC determined for men in the San Francisco-Oakland area appears to be systematically lower than the corresponding estimates for Connecticut or Detroit areas. Analogous distributions are observed for the hazard functions of LC determined for women in these areas. Based on these observations, we hypothesized that the risk factors of LC, associated with geographical area, uniformly influence the values of the age-specific hazard functions.

To test this hypothesis, we used the age-specific hazard function of the San Francisco-Oakland area as a standard to estimate the relative age-specific hazards, $r_{1|0}^{*}(t_{i})=h_{1}^{*}(t_{i})/h_{0}^{*}(t_{i})$ (and their *SE*), for Connecticut vs. the San Francisco-Oakland and the relative age-specific hazards, $r_{2|0}^{*}(t_{i})=h_{2}^{*}(t_{i})/h_{0}^{*}(t_{i})$ (and their *SE*), for Detroit vs. the San Francisco-Oakland area. The obtained estimates of the relative hazards (and their *SE*) of LC for men and women are given in Tables 3 and 4, correspondingly.

To perform graphical analysis of the estimates of the age-specific relative hazards, $r_{1|0}^{*}(t_{i})$ and $r_{2|0}^{*}(t_{i})$, we used 95% confidence intervals (95% CI), $r_{1|0}^{*}(t_{i})\pm 1.96\cdot \text{SE}[r_{1|0}^{*}(t_{i})]$ and $r_{2|0}^{*}(t_{i})\pm 1.96\cdot \text{SE}[r_{2|0}^{*}(t_{i})]$. Preliminary analysis showed that the estimates of the age-specific relative hazards are slightly fluctuated near certain constants depending on the considered geographical area and gender. To determine these constants, we applied the linear regression analysis. In this case, the most efficient estimates of the corresponding constants can be obtained by formula (8). We determined the estimates of the averaged relative hazards of LC in the Connecticut vs. San Francisco-Oakland areas, $R_{1|0}^{*}$, and in the Detroit vs. San Francisco-Oakland areas, $R_{2|0}^{*}$. The *SE* of the corresponding estimates was calculated by formula (9).

Outliers (i.e. those points which have large influence on the resulting fit) were excluded by the standard procedures of the linear regression analysis.^14^ After omitting these outliers, the estimates of the constants were recomputed.

Calculations showed that for men living in Connecticut vs. San Francisco-Oakland, the estimate of the averaged relative hazard (±*SE*) of LC is 1.31 ± 0.02, while for men living in Detroit vs. San Francisco-Oakland this estimate is 1.53 ± 0.02. Analogous calculations suggest that for women living in Connecticut vs. San Francisco-Oakland, the averaged relative hazard is 1.22 ± 0.02, while for women living in Detroit vs. San Francisco-Oakland this hazard is 1.32 ± 0.02.

**Figure 3.**
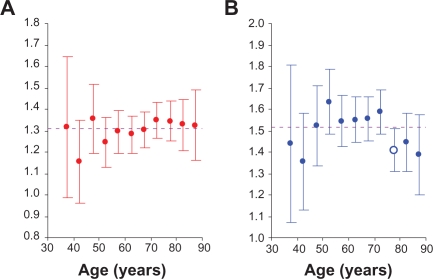
The estimates of the age-specific relative hazards in white men: (**A**) for Connecticut vs. San Francisco-Oakland and (**B**) for Detroit vs. San Francisco-Oakland. Error bars indicate 95% confidence intervals. Open circles indicate outliers. Dashed line indicates averaged hazard.

**Figure 4.**
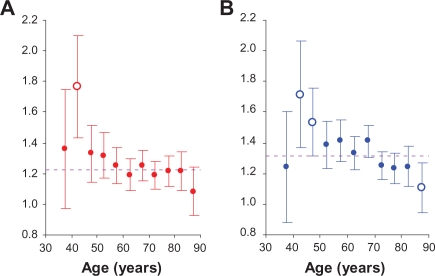
The estimates of the age-specific relative hazards in white women: (**A**) for Connecticut vs. San Francisco-Oakland and (**B**) for Detroit vs. San Francisco-Oakland. Error bars indicate 95% confidence intervals. Open circles indicate outliers. Dashed line indicates averaged relative hazard.

In Figure 3, panel (A) shows the graph of the relative hazards with their 95% CI, $r_{1|0}^{*}(t_{i})\pm 1.96\cdot \text{SE}[r_{1|0}^{*}(t_{i})]$, for white men in Connecticut vs. San Francisco-Oakland. Panel (B) of this figure shows the relative hazards with 95% CI, $r_{2|0}^{*}(t_{i})\pm 1.96\cdot \text{SE}[r_{2|0}^{*}(t_{i})]$, for men in Detroit vs. San Francisco-Oakland. Analogously, panels A and B in Figure 4 show the relative hazards with 95% CI, for white women. On these panels, the horizontal line indicates the average of the relative hazards and error bars indicate the 95% CI.

Assuming that the estimate of the averaged relative hazard is equal to the mathematical expectation of this estimator, the estimates of the relative hazards can be compared with the averaged relative hazard. When the 95% CI of the relative hazard intersects with the corresponding averaged relative hazard, this relative hazard can be considered as statistically indistinguishable from the averaged value.

Analysis of Figures 3 and 4 suggests that the age-specific relative hazards of LC are nearly constant and depend on the geographical areas and gender. In fact, data presented in Table 3 (after excluding one outlier) show that the risk of LC in Connecticut vs. San Francisco-Oakland is about 1.3 times higher for men, whereas for women, it is about 1.2 times higher. Analogously, data in Table 4 (after excluding outliers) show that for men in Detroit vs. San Francisco-Oakland this risk is about 1.5 times higher, while for women, it is about 1.3 times higher. In this connection, it should be mentioned that the trends appearing on Figures 3 and 4 are much exaggerated. This is because the scale of the *x* axis on these figures is about 100 times smaller than the scale for the *y* axis. Performed regression analysis showed, however, that slopes of the linear regression lines for men in Connecticut vs. San Francisco-Oakland (Fig. 3A) and Detroit vs. San Francisco-Oakland (Fig. 3B) are 0.0023 (*SE* of 0.0009) and 0.0014 (*SE* of 0.0020), correspondingly. Analogous slopes of the linear regression lines for women in Connecticut vs. San Francisco-Oakland (Fig. 4A) and Detroit vs. San Francisco-Oakland (Fig. 4B) are −0.0038 (*SE* of 0.0012) and −0.0056 (*SE* of 0.0023), correspondingly. We also found that even when outliers are not excluded, the slopes for men and women do not exceed 0.008 (i.e. the values of slopes are always near zero). This suggests that the age-specific relative hazards of LC are nearly constant.

**Figure 5.**
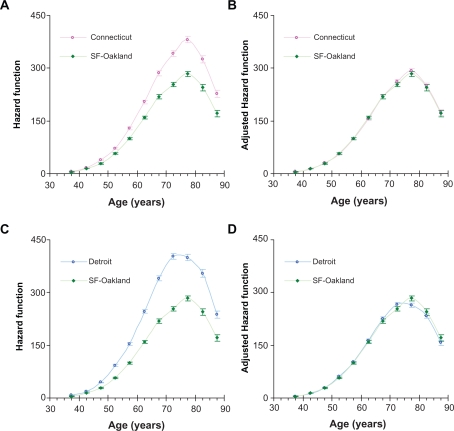
Comparison of age-specific hazard functions of lung cancer in white men unadjusted (**A** and **C**) and adjusted (**B** and **D**) for geographical location. Error bars indicate standard errors. **A**) Unadjusted hazard functions in Connecticut and San Francisco-Oakland. **B**) Adjusted hazard functions in Connecticut and San Francisco-Oakland (with the San Francisco-Oakland area as the standard). **C**) Unadjusted hazard functions in Detroit and San Francisco-Oakland area. **D**) Adjusted hazard functions in Detroit and San Francisco-Oakland (with the San Francisco-Oakland area as the standard).

**Figure 6.**
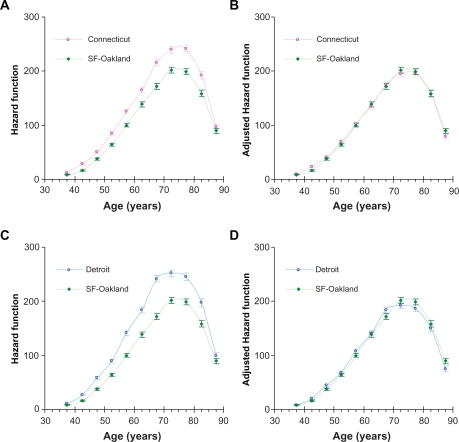
Comparison of age-specific hazard functions of lung cancer in white women unadjusted (**A** and **C**) and adjusted (**B** and **D**) for geographical location. Error bars indicate standard errors. **A**) Unadjusted hazard functions in Connecticut and San Francisco-Oakland. **B**) Adjusted hazard functions in Connecticut and San Francisco-Oakland (with the San Francisco-Oakland area as the standard). **C**) Unadjusted hazard functions in Detroit and San Francisco-Oakland area. **D**) Adjusted hazard functions in Detroit and San Francisco-Oakland (with the San Francisco-Oakland area as the standard).

Based on this analysis, we suggest that the risk factors of LC, associated with the geographical area, uniformly influence the values of the age-specific hazard functions. This can be illustrated by Figures 5 and 6 showing that after adjustments by the corresponding averaged relative hazard, the shapes of the age-specific hazard functions for white men and women living in Connecticut and Detroit are almost identical to the corresponding age-specific hazard functions for white men and women living in the San Francisco-Oakland area. For Connecticut and Detroit, adjustments of their hazard functions to the hazard function of the San Francisco-Oakland area were performed by dividing the hazard function values by the corresponding values of the averaged relative hazard.

## Conclusion

In this work, we proposed an efficient computing procedure for estimation of the age-specific hazard functions in the LLAPC model. This procedure is based on the novel approach for analysis of time period and birth cohort effects on the distribution of the age-specific cancer incidence rates, developed in our previous work.^11^

The procedure proposed in the present work allows one to estimate the age-specific hazard functions for populations with different exposures to a given categorical risk factor. The ratios of hazard functions for populations with different exposures to a given categorical risk factor are used for characterizing relative age-specific hazards of cancers.

As a proof-of-concept that this procedure can be used to evaluate the influence of categorical risk factors on the age-specific hazard functions, we estimated LC risk for populations living in different geographical areas. For this purpose, we utilized data on the LC incidence rates in white men and women, collected in the San Francisco-Oakland, Connecticut and Detroit areas during 1975–2004.

We have found that the risks of LC in white men and women, associated with living in these geographical areas, differ in amplitude but the overall shape of these functions are similar, i.e. the geographical area risk factors influence the LC age-specific hazard functions in approximately the same manner in all ages. We have shown that in white men the averaged relative hazard of LC in Connecticut vs. San Francisco-Oakland is 1.31 ± 0.02, while in Detroit vs. San Francisco-Oakland this relative hazard is about 1.53 ± 0.02. In white women, analogous relative hazards in Connecticut vs. San Francisco-Oakland and Detroit vs. San Francisco-Oakland are 1.22 ± 0.02 and 1.32 ± 0.02, correspondingly.

We suggest that the proposed computing procedure can be used for assessing hazard functions for other categorical risk factors, such as gender, race, lifestyle, diet, obesity, etc.

## Figures and Tables

**Table 1.** Estimates of the age-specific hazard functions, $h_{0}^{*}(t_{i})$, $h_{1}^{*}(t_{i})$, and $h_{2}^{*}(t_{i})$, and their standard errors (*SE*) for white men in three geographical areas: San Francisco-Oakland, Connecticut, and Detroit.

**Age group**		**Geographical areas**					
		**San Francisco-Oakland**		**Connecticut**		**Detroit**	
***i***	***t_i_***	${\text{h}}_{0}^{*}({\text{t}}_{\text{i}})$	***SE***	${\text{h}}_{1}^{*}({\text{t}}_{\text{i}})$	***SE***	${\text{h}}_{2}^{*}({\text{t}}_{\text{i}})$	***SE***
8	37.5	5.11	0.50	6.72	0.62	7.35	0.64
9	42.5	14.34	0.92	16.58	1.02	19.43	1.12
10	47.5	28.90	1.39	39.10	1.69	44.07	1.79
11	52.5	56.89	2.11	70.97	2.38	93.04	2.82
12	57.5	99.87	3.10	129.20	3.51	154.19	3.91
13	62.5	158.96	4.40	203.60	4.82	246.40	5.47
14	67.5	218.55	5.68	285.37	6.21	340.10	7.06
15	72.5	253.56	6.60	341.70	7.29	402.29	8.25
16	77.5	283.49	7.95	381.33	8.65	399.53	9.14
17	82.5	245.25	9.08	325.40	9.96	354.44	11.19
18	87.5	171.04	8.72	226.73	9.62	236.97	10.93

**Table 2.** Estimates of the age-specific hazard functions, $h_{0}^{*}(t_{i})$, $h_{1}^{*}(t_{i})$, and $h_{2}^{*}(t_{i})$, and their standard errors (*SE*) for white women in three geographical areas: San Francisco-Oakland, Connecticut, and Detroit.

**Age group**		**Geographical areas**					
		**San Francisco-Oakland**		**Connecticut**		**Detroit**	
***i***	***t_i_***	${\text{h}}_{0}^{*}({\text{t}}_{\text{i}})$	***SE***	${\text{h}}_{1}^{*}({\text{t}}_{\text{i}})$	***SE***	${\text{h}}_{2}^{*}({\text{t}}_{\text{i}})$	***SE***
8	37.5	8.55	0.92	11.65	1.18	10.63	1.09
9	42.5	15.90	1.25	28.08	1.89	27.29	1.81
10	47.5	37.96	2.09	50.51	2.59	58.29	2.79
11	52.5	64.57	2.79	85.15	3.39	89.58	3.44
12	57.5	99.84	3.61	124.95	4.14	141.42	4.47
13	62.5	138.60	4.42	165.12	4.79	184.48	5.18
14	67.5	171.63	5.04	214.92	5.58	242.01	6.10
15	72.5	201.87	5.68	239.67	5.90	252.02	6.19
16	77.5	198.99	5.93	241.90	6.20	245.54	6.50
17	82.5	158.51	6.27	192.88	6.75	197.33	7.22
18	87.5	89.46	4.77	96.76	4.74	99.12	5.41

**Table 3.** Estimates of the age-specific hazard function ratios and their standard errors (*SE*) for Connecticut vs. San Francisco-Oakland.

**Connecticut vs. San Francisco-Oakland**					
**Age intervals**		**Men**		**Women**	
***i***	***t_i_***	${\text{r}}_{1|0}^{*}({\text{t}}_{\text{i}})$	***SE***	${\text{r}}_{1|0}^{*}({\text{t}}_{\text{i}})$	***SE***
8	37.5	1.32	0.33	1.36	0.39
9	42.5	1.16	0.19	1.77	0.33
10	47.5	1.35	0.16	1.33	0.19
11	52.5	1.25	0.12	1.32	0.15
12	57.5	1.29	0.10	1.25	0.12
13	62.5	1.28	0.09	1.19	0.10
14	67.5	1.31	0.08	1.25	0.10
15	72.5	1.35	0.09	1.19	0.09
16	77.5	1.35	0.09	1.22	0.10
17	82.5	1.33	0.12	1.22	0.13
18	87.5	1.33	0.17	1.08	0.16

**Table 4.** Estimates of the age-specific hazard function ratios and their standard errors (*SE*) for Detroit vs. San Francisco-Oakland.

**Age intervals**		**Detroit vs. San Francisco-Oakland**			
		**Men**		**Women**	
***i***	***t_i_***	${\text{r}}_{2|0}^{*}({\text{t}}_{\text{i}})$	***SE***	${\text{r}}_{2|0}^{*}({\text{t}}_{\text{i}})$	***SE***
8	37.5	1.44	0.37	1.24	0.36
9	42.5	1.35	0.23	1.72	0.35
10	47.5	1.52	0.19	1.54	0.22
11	52.5	1.64	0.15	1.39	0.16
12	57.5	1.54	0.12	1.42	0.13
13	62.5	1.55	0.11	1.33	0.11
14	67.5	1.56	0.10	1.41	0.11
15	72.5	1.59	0.10	1.25	0.09
16	77.5	1.41	0.10	1.23	0.10
17	82.5	1.45	0.14	1.24	0.13
18	87.5	1.39	0.19	1.11	0.17
